# Insufficient serum L-ficolin is associated with disease presence and extent of pulmonary *Mycobacterium avium* complex disease

**DOI:** 10.1186/s12931-019-1185-9

**Published:** 2019-10-21

**Authors:** Tomofumi Kobayashi, Koji Kuronuma, Atsushi Saito, Kimiyuki Ikeda, Shigeru Ariki, Atsushi Saitou, Mitsuo Otsuka, Hirofumi Chiba, Satoshi Takahashi, Motoko Takahashi, Hiroki Takahashi

**Affiliations:** 1 Department of Respiratory Medicine and Allergology, Sapporo Medical University School of Medicine, South-1 West-16, Chuo-ku, Sapporo, 060-8556 Japan; 2 Department of Biochemistry, Sapporo Medical University School of Medicine, South-1 West-16, Chuo-ku, Sapporo, 060-8556 Japan; 3 Department of Infection Control and Laboratory Medicine, Sapporo Medical University School of Medicine, South-1 West-16, Chuo-ku, Sapporo, 060-8556 Japan

**Keywords:** *Mycobacterium avium* complex, L-ficolin, Ficolin-2, Collectin, Biomarker

## Abstract

**Background:**

The incidence of infectious disease caused by nontuberculous mycobacteria is increasing worldwide. Pulmonary *Mycobacterium avium* complex (MAC) disease is difficult to treat with chemotherapy, and its mechanism of infection, infection route, disease onset, and severity remain unknown. Ficolins are oligomeric defense lectins. L-ficolin plays an important role in innate immunity. This study’s aim was to identify L-ficolin’s role in patients with pulmonary MAC disease.

**Methods:**

Between April 2011 and September 2017, 61 Japanese patients with pulmonary MAC disease were seen at our hospital. A control group, comprising 30 healthy individuals, without respiratory disease were enrolled in our study. The relationship between serum L-ficolin levels and disease severity was assessed, and L-ficolin’s antibacterial role was examined.

**Results:**

Serum L-ficolin levels were significantly lower in patients with pulmonary MAC disease than in healthy subjects (1.69 ± 1.27 μg/ml vs. 3.96 ± 1.42 μg/ml; *p* < 0.001). The cut-off value, based on receiver operating characteristic (ROC) analysis results, was 2.48 μg/ml (area under the curve (AUC) 0.90, sensitivity and specificity 83.6 and 86.7%, respectively). Serum L-ficolin levels were significantly lower in the patients with nodular bronchiectatic type disease compared with the patients with fibrocavitary type disease and were lower in the high-resolution computed tomography high-scoring group compared with low-scoring group. An in vitro analysis showed that purified recombinant L-ficolin bound to *M. avium* and its major cell wall component, lipoarabinomannan, in a concentration-dependent manner. In addition, recombinant L-ficolin suppressed *M. avium* growth in a concentration-dependent manner.

**Conclusions:**

Insufficient serum L-ficolin is associated with disease progression in pulmonary MAC disease, and the level of serum L-ficolin is a possible biomarker.

**Trial registration:**

This study is registered with UMIN (UMIN000022392).

## Background

Nontuberculous mycobacteria (NTM) have been seen as environmental organisms of limited clinical relevance, overshadowed by the more aggressive *Mycobacterium tuberculosis*. However, chronic pulmonary disease, caused by NTM, is increasing in Japan because of an aging population. In 2014, the NTM incidence rate in Japan was 14.7 per 100,000 based on national surveillance data [1]. *Mycobacterium avium* complex (MAC) organisms are the major causative bacteria of NTM infection in patients who do not have cystic fibrosis; these organisms are difficult to treat with chemotherapy. Pulmonary MAC disease is responsible for approximately 80% of the NTM cases in Japan. A systematic review indicated a high mortality rate among patients with pulmonary MAC [2].

Older age and female sex are well-known risk factors for NTM infection. Having a low body mass index (BMI) also appears to be a risk factor for NTM infection; higher BMI has a protective effect. Substantial heterogeneity was found among male patients, with the presence of cavity disease and high comorbidity levels predicting worse outcomes. In terms of molecular pathology, the adipokines leptin and adiponectin may be responsible for the presence of NTM disease [3–6]. Severe vitamin D deficiency also appears to be associated with NTM disease [7].

Innate immune molecules can suppress the early stages of infection via mechanisms mediated by many different recognition systems and effectors, including the complement system. Pulmonary surfactant protein (SP-)A, SP-D, mannose-binding lectin (MBL), and ficolins are all types of complement lectins, capable of recognizing microbial carbohydrates and activating the lectin complement pathway via a mechanism similar to that of the classical pathway.

Ficolins are oligomeric defense, proteins assembled from collagen-like stalks and fibrinogen-like domains that can sense danger signals, such as pathogens and apoptotic cells. Three members of the ficolin family have been identified and characterized in humans: M-ficolin (ficolin-1), L-ficolin (ficolin-2), and H-ficolin (ficolin-3, or Hakata antigen) [8–10]. Human L-ficolin and H-ficolin are mainly expressed in the liver and present in the circulation as serum lectins.

Human L-ficolin has lectin-like activity for N-acetylglucosamine (GlcNAc), lipopolysaccharides, 1,3-beta-D-glucan, lipoteichoic acid, and various acetylated compounds [11–13].

L-ficolin’s fibrinogen-like domain forms a globular structure, similar to the carbohydrate-recognition domains of MBL, SP-A, and SP-D, and binds to sugar structures. Ficolins also show opsonic activity via the binding of their collagen-like domain to macrophages [14].

L-ficolin is said to specifically bind to some clinically important microorganisms (group B streptococcus, *Streptococcus pneumoniae,* and *M. tuberculosis*), act as an opsonin, and promote microbe elimination through the elicitation of phagocytosis or the activation of the lectin pathway of the complement system in in vitro tests [15–19].

So far, we have clarified that SP-A and SP-D play important roles in MAC infections [20, 21]. Conversely, L-ficolin’s role in MAC infections remains completely unknown. This study’s aim was to identify L-ficolin’s role in MAC infections. Our findings support the notion that L-ficolin plays a role in the pathogenesis of MAC infections and suggest that L-ficolin may be a novel biomarker of MAC infection as well as a therapeutic target.

## Methods

### Participants

This case-control study was conducted at a single center, the Sapporo Medical University Hospital, Sapporo, Japan, between April 2011 and September 2017. The hospital’s ethics board approved this study (approval number 272–168), and all participants gave written informed consent.

Patients with NTM infection were included if they visited or were admitted to the hospital, and NTM infection was diagnosed based on the 2007 diagnostic criteria of the American Thoracic Society/Infectious Disease Society of America [22]. Exclusion criteria were the patient’s inability to give informed consent and an NTM infection not caused by MAC. The control group participants included healthy volunteers who showed no evidence of respiratory disease following a Shihoro-cho health check.

### Measurement of serum L-ficolin concentration

Serum L-ficolin levels were measured using an ELISA Kit (Hycult Biotech Inc., PA, USA) according to the manufacturer’s instructions.

### High-resolution computed tomography (HRCT) scoring

Within 3 months of entry into the study, thin slice (1 mm) HRCT images were obtained from the participants at 10 mm intervals from the lung apex to the base during suspended full inspiration with the participant in the supine position. All HRCT data were reconstructed using a high spatial frequency algorithm and displayed on the lung parenchymal window (level, − 700 Hounsfield units (HU); width, 1500 HU). The HRCT images were scored for the severity and the extent of the pulmonary MAC infection using the modified scoring system of Fowler et al. [23]. The summary of the HRCT scoring of the whole lung is shown in Additional file 1: Table S1A. To identify the differences in serum L-ficolin levels by severity of MAC disease, MAC patients were separated into two groups according to the HRCT score. The HRCT low-score group was ≤6 and the high-score group was ≥7.

### Binding of L-ficolin to *M. avium*

MycoBroth and 7H11-C agar plates were purchased from Kyokuto Pharmaceutical (Tokyo, Japan). *M. avium* cultures were purchased from ATCC (No. 700898). This strain was the *M. avium* subspecies *hominissuis,* which is a major human MAC subtype in Japan [24]. Another *M. avium* strain was clinically isolated from the sputum of an infected patient. The bacteria were cultured in MycoBroth and suspended in phosphate-buffered saline. The concentration of the bacterial suspension was determined by measuring the absorbance at 600 nm.

A suspension of UV-killed *M. avium* was coated onto microtiter wells and then dried. After the wells were washed, the bacteria were incubated with L-ficolin at 37 °C for 1 h. The wells were washed and then incubated with anti-L-ficolin antibodies and secondary antibodies at 37 °C for 1 h. The binding of L-ficolin to *M. avium* was detected by measuring the absorbance at 492 nm.

### Surface plasmon resonance analysis

Sonicated lipoarabinomannan (LAM) was immobilized on an HPA sensor chip of the Biacore 3000 system (Biacore, Uppsala, Sweden) according to the manufacturer’s specifications. L-ficolin was injected at a flow rate of 30 μl/min. Sensorgrams of the interactions obtained using various concentrations of L-ficolin were analyzed using the BIAevaluation program.

### Growth inhibition assay

*M. avium* (2000 CFU) was cultured in MycoBroth at 37 °C for 96 h in the presence or absence of L-ficolin or other lectins. At 0, 8, 24, 48, and 96 h an aliquot of the culture was withdrawn and spread onto a 7H11-C agar plate. The plates were incubated at 37 °C for 48 h, and the number of colonies on the plates was then counted.

### Statistical analysis

All statistical analyses were performed using JMP 13.0 (SAS Institute, Cary, NC, USA) and GraphPad Prism v7 software (GraphPad, Inc., San Diego, CA, USA). Student’s t-test or one-way analysis of variance (ANOVA) with post-hoc Tukey test were used to analyze continuous parametric data.

For all analyses, a *p* value < 0.05 was considered statistically significant.

## Results

This study included 61 patients with pulmonary MAC and 30 healthy volunteers in the control group. The participants’ characteristics are shown in Table 1.

**Table 1:** Study participants’ characteristics

	Control group (*n* = 30)	MAC Patients (*n* = 61)	*p* value
L-Ficolin (μg/ml)	3.96 ± 1.42	1.69 ± 1.27	*p* < 0.001
Age (years)	45.7 ± 15.5	70.9 ± 8.9	*p* < 0.001
Median (years)(min–max)	46.5 (20–78)	71 (40–89)	*p* < 0.001
Sex (male/female)	23/7	11/50	*p* < 0.001
History of smoking (never / < 20 packs / > 20 packs)	19/10/1	43/11/7	–
BMI (kg/m^2^)	23.9 ± 3.6	20.3 ± 3.0	*p* < 0.001
WBC (/μl)	5350 ± 1795	5832 ± 1662	*p* = 0.208
CRP (mg/dl)	0.08 ± 0.08	0.30 ± 0.56	*p* = 0.032
Alb (g/dl)	4.52 ± 0.27	3.87 ± 0.35	*p* < 0.001
SP-A (ng/ml)	29.5 ± 11.3	37.0 ± 16.5	*p* = 0.027
SP-D (ng/ml)	60.0 ± 37.3	117.5 ± 80.5	*p* < 0.001

Data are presented as mean ± SD. *p* values were calculated using the Student’s t-test

*Abbreviations BMI* body mass index, *WBC* white blood cells, *CRP* C-reactive protein, *Alb* albumin, *SP-A* surfactant protein A, *SP-D* surfactant protein D

Patients with MAC were aged 70.9 ± 8.9 years on average and included 11 males (18.0%) and 50 females (82.0%); this was different from the control group but comparable to Japanese MAC surveillance data [1]. The MAC patients’ average BMI was 20.3 ± 3.0 kg/m^2^, which was lower than that for individuals in the control group. Among the MAC patients, 43 (70.5%) had never smoked. Although white blood cell (WBC) counts were not significantly elevated, C-reactive protein (CRP) and serum SP-A, and SP-D levels were significantly higher in patients with MAC compared with those in the control group, suggesting an inflammatory reaction in the lungs of patients with pulmonary MAC disease.

The bacteriologically detected species of NTM were *M. avium* (*n* = 47, 77.0%), *M. intracellulare* (*n* = 8, 13.1%), and mixed infections of MAC and other mycobacteria (*n* = 6, 9.8%) (Table 2). Anti-MAC antibodies were detected in 43 MAC patients (70.5%). The positivity rate of anti-MAC antibodies in our study was similar to that of Japanese MAC patients described in a previous study [25]. Twelve participants (19.7%) were undergoing treatment with immunosuppressants because of comorbidity. Four patients (6.6%) tested positive by interferon-gamma releasing assay (IGRA). Combination chemotherapy was administered to 28 MAC patients (45.9%) while they were participating in this study.

**Table 2:** The status of patients with MAC (*n* = 61)

Type of bacteria	*M.avium*	47
	*M.intracellulare*	8
	*M.avium* + *M.intracellulare*	2
	*M.avium* + *M.chelonae*	1
	*M.avium* + *M.abscessus*	1
	*M.avium* + *M.scrofulaceum*	1
	*M.intracellulare* + *M.fortuitum*	1
HRCT findings	NB type/NB + FC type/FC type	47/5/7
MAC-Ab (antibody titer)	positive/negative	43 (0.7–87.6) / 18 (< 0.6)
Treatment	untreated/undergoing treatment/post-treatment	33/21/7
Immunosuppressive drug	positive/negative	12/49
IGRA (QFT or T-SPOT)	positive/negative/undeterminable or unknown	4/51/6

Data are presented as the number of patients

*Abbreviations*: *HRCT* high-resolution computed tomography, *NB type* nodular bronchiectatic type, *FC type* fibrocavitary type, *IGRA* interferon-gamma releasing assay

### Comparison between the MAC and the control groups

**Fig. 1:**
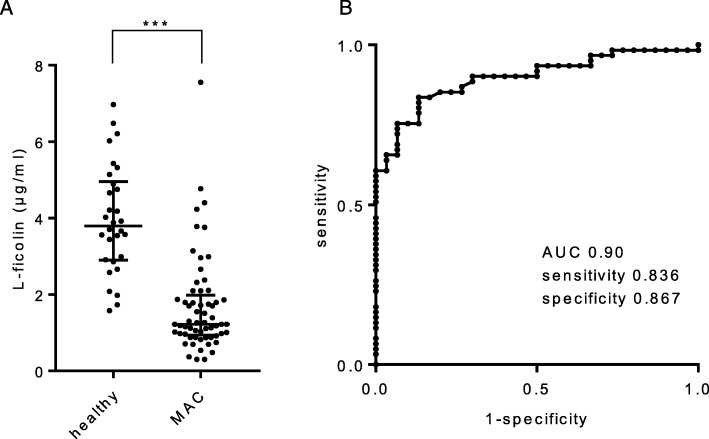
**a** Difference in serum L-ficolin levels between the *Mycobacterium avium* complex (MAC) patients and the healthy participants. Lines express the mean ± SD. Student’s t-test, *** *p* < 0.001. **b** Receiver operating characteristic curve constructed between MAC patients and healthy participants

The serum L-ficolin level was quantified using an ELISA kit. Serum L-ficolin was 1.69 ± 1.27 μg/ml in MAC patients compared with 3.96 ± 1.42 μg/ml in control subjects (Fig. 1a, *p* < 0.001). A receiver operating characteristic (ROC) curve constructed between the MAC patients and healthy control participants revealed an area under the curve (AUC) of 0.90, a sensitivity of 83.6%, and a specificity of 86.7% when the cut-off for serum L-ficolin was set at 2.48 μg/ml (Fig. 1b).

### Radiographic disease extent and L-ficolin

**Fig. 2:**
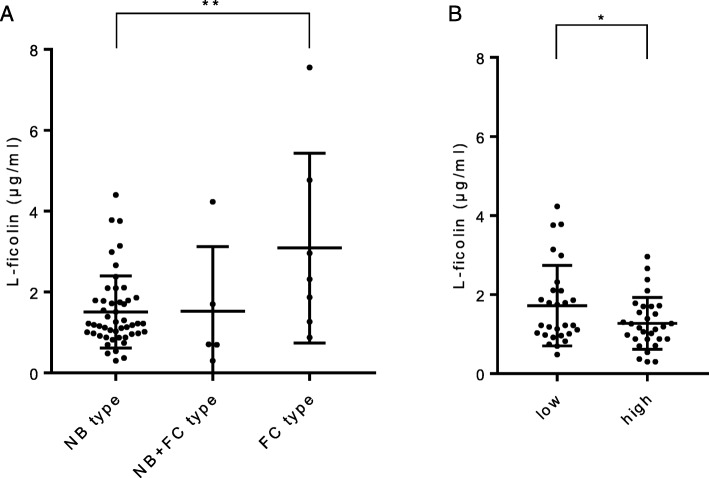
**a** Differences in serum L-ficolin levels by type of MAC disease. NB type, nodular bronchiectatic type; FC type, fibrocavitary type. Lines express the mean ± SD. One-way ANOVA with post-hoc Tukey test, ** *p* < 0.01. **b** Differences in serum L-ficolin levels by severity of MAC disease. MAC patients were separated into two groups by HRCT scoring. The HRCT low-score group was ≤6 and the high-score group was ≥7. Lines express the mean ± SD. Student’s t-test, * *p* < 0.05

From the HRCT findings in MAC patients, the mean serum L-ficolin level in those patients with fibrocavitary type (FC type) was 3.09 ± 2.35 μg/ml, similar to the level seen in the healthy control group (Fig. 2a). However, the mean serum L-ficolin level in the patients with the nodular bronchiectatic type (NB type) was 1.51 ± 0.89 μg/ml, significantly lower than the level in the patients with the FC type (*p* < 0.01). Compared with patients with the FC type, the patients with the NB type also showed lower levels of other parameters, such as WBC, CRP, albumin, and SP-A, indicating that the NB type could elicit a lower inflammatory response (Table 3). To evaluate the disease progression of pulmonary MAC, we scored the HRCT findings using a scoring system (Additional file 1: Table S1A). The average HRCT score was 6.25 ± 3.30 (Additional file 1: Table S1B). Serum L-ficolin levels in the high-score group were lower than those in the low-score group (Fig. 2b, *p* < 0.05).

**Table 3:** HRCT types and laboratory data for patients with MAC

	NB type (*n* = 49)	NB + FC type (*n* = 5)	FC type (*n* = 7)	*p* value
L-Ficolin (μg/ml)	1.51 ± 0.89	1.53 ± 1.60	3.09 ± 2.35	*p* = 0.006
Age (years)	70.7 ± 8.2	72.0 ± 5.1	71.6 ± 15.6	*p* = 0.934
BMI (kg/m^2^)	20.5 ± 3.0	17.7 ± 1.9	21.0 ± 3.6	*p* = 0.120
WBC (/μl)	5557 ± 1516	6680 ± 1654	7157 ± 2036	*p* = 0.026
CRP (mg/dl)	0.16 ± 0.23	0.38 ± 0.61	1.27 ± 1.09	*p* < 0.001
Alb (g/dl)	3.95 ± 0.29	3.60 ± 0.16	3.47 ± 0.50	*p* = 0.001
SP-A (ng/ml)	33.6 ± 14.2	50.6 ± 20.0	51.3 ± 19.0	*p* = 0.003
SP-D (ng/ml)	109.5 ± 74.9	186.7 ± 118.3	124.7 ± 77.4	*p* = 0.119

Data are presented as mean ± SD. *p* values were calculated using the one-way ANOVA with post-hoc Tukey test

### Direct interaction between *M. avium* and L-ficolin

**Fig. 3:**
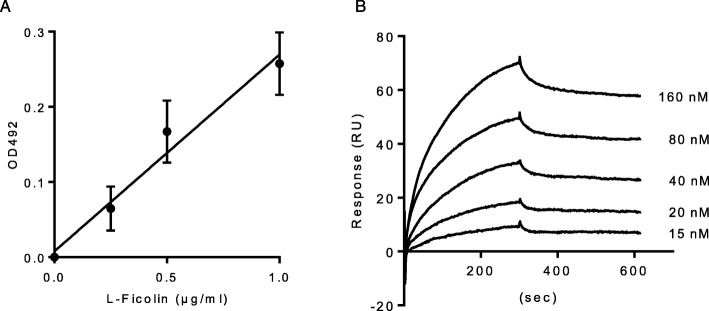
The binding of recombinant human L-ficolin to *M. avium*. **a** The indicated concentrations of recombinant L-ficolin were incubated with clinically isolated *M. avium* coated onto microtiter wells. Bound L-ficolin was detected at OD 492 nm. The data show the mean ± SD of three independent experiments. **b** The binding parameters of recombinant L-ficolin with *M. avium* were determined by surface plasmon resonance analysis. Sensorgrams for the binding of recombinant L-ficolin to *M. avium* immobilized on a sensor chip were overlaid at various concentrations of L-ficolin

We first confirmed the binding of recombinant L-ficolin to *M. avium* coated onto microtiter wells. L-ficolin bound to *M. avium* in a concentration-dependent manner (Fig. 3a). To determine whether the ligand for L-ficolin was LAM, surface plasmon resonance sensor analysis was performed (Fig. 3b). The passage of L-ficolin at various concentrations over LAM immobilized on a sensor chip yielded an association rate constant, ka, of 2.41 × 10^5^ M^− 1^ s^− 1^ and a dissociation rate constant, kd, of 2.16 × 10^− 4^ s^− 1^, giving a subsequent dissociation constant, KD (kd/ka), of 8.96 × 10^− 10^ M. These results indicate that L-ficolin binds to *M. avium* and that the ligand targeted by L-ficolin might be LAM.

### Complement lectins inhibit the growth of *M. avium*

**Fig. 4:**
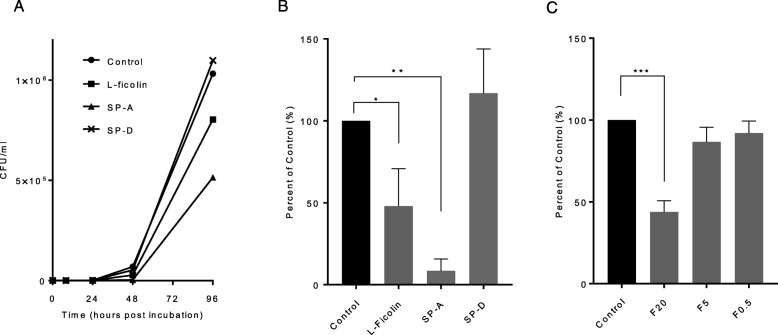
**a** L-ficolin and SP-A attenuate the growth of *M. avium*. *M. avium* from ATCC was cultured at 37 °C in the presence of the indicated concentrations of L-ficolin, SP-A, and SP-D. The number of CFUs are presented. **b** Percentage of untreated control *M. avium* growth after 48 h with L-ficolin, SP-A, and SP-D. The data show the mean ± SD of three independent experiments. One-way ANOVA with post-hoc Tukey test, * *p* < 0.05, ** *p* < 0.01. **c** Percentage of untreated control *M. avium* growth after 48 h at various L-Ficolin concentrations. The data show the mean ± SD of three independent experiments. One-way ANOVA with post-hoc Tukey test, *** *p* < 0.001. F20, 20 μg/ml L-ficolin; F5, 5 μg/ml L-ficolin; F0.5, 0.5 μg/ml L-ficolin

We examined whether L-ficolin affected the growth of *M. avium* in vitro (Fig. 4a). SP-A and SP-D were used as positive and negative controls, respectively. Cultures were started at 2000 CFU/ml, and no significant differences were observed among the samples between hours 0 and 24. However, L-ficolin clearly inhibited *M. avium* growth after 48 h compared with the control and SP-D samples (Fig. 4b). Furthermore, L-ficolin 0.5–20 μg/ml inhibited *M. avium* growth after 48 h in a concentration-dependent manner, and growth under conditions with 20 μg/ml L-ficolin was significantly suppressed compared with the untreated control growth (Fig. 4c).

## Discussion

This is the first study to investigate L-ficolin serum levels in relation to clinical pulmonary MAC disease. We found a significant difference in serum L-ficolin levels between MAC patients and control subjects. Also, serum L-ficolin levels were found to be related to the severity of MAC disease. Additionally, it was revealed that L-ficolin bound directly to *M. avium* and inhibited its growth, suggesting that L-ficolin activity worked as a host defense against *M. avium* infection.

MAC patients with cavity type disease have higher mortality rates than those without cavity type disease [25]. From the data obtained by HRCT analysis of MAC types, the FC type exhibits a stronger inflammatory response than the NB type. The characteristics of patients with NB type MAC also had less inflammation.

Serum L-ficolin levels in MAC patients showed high sensitivity and specificity (83.6 and 86.7%, respectively), and indicated a negative correlation with the extent of the disease. Kitada et al. proved the usefulness of ELISA kits in diagnosing MAC [26]. Higher human serum antibody levels to glycopeptidolipid core antigen showed high sensitivity and specificity (84.3 and 100%, respectively), and revealed a positive correlation with the extent of disease. Low serum L-ficolin levels are associated with several lung diseases. The serum L-ficolin levels of patients with pulmonary tuberculosis has been shown to be lower than that of healthy individuals [27]. L-ficolin can bind to *M. tuberculosis* and defend against *M. tuberculosis* infection by activating innate immune responses. Insufficient serum L-ficolin levels have been seen in patients with severe community-acquired pneumonia [28]. Patients with bronchiectasis also have very low L-ficolin levels [29, 30]. Insufficient L-ficolin is not specific for pulmonary MAC, but suggests existing chronic or infectious lung diseases.

According to the criteria for MAC, single and mixed mycobacterial infections were included, as shown in Table 2. When we exclude the four subjects with mixed infections, 57 cases of MAC also had significantly low L-ficolin levels (1.65 ± 1.28 μg/ml) and similar correlations of L-ficolin with the disease extent (data not shown).

Ficolins bind to a variety of ligands on various pathogens. L-ficolin’s fibrinogen-like domain forms a globular structure, similar to the carbohydrate-recognition domains of MBL, SP-A, and SP-D, and binds to sugar structures. According to our binding analysis, L-ficolin’s binding to *M. avium* was specific but not completely dissociated by EDTA (data not shown). Previous reports indicated that the ligand for SP-D is LAM [20]. Our surface plasmon resonance sensor analysis indicated that L-ficolin attached to LAM; however, LAM did not completely inhibit L-ficolin’s binding to *M. avium* when using ELISA methods (data not shown). As a result, we could not determine if there were one-to-one binding sites. Additional experiments to identify the ligand for L-ficolin will be necessary in the future.

Previous studies have shown that complement lectins inhibit the growth of mycobacteria. Ariki et al. indicated that SP-A, but not SP-D, strongly inhibited *M. avium* growth without complement activation [20]. We revealed that human L-ficolin also directly inhibited *M. avium* growth. L-ficolin and SP-A form a bouquet-like structure consisting of six trimeric subunits. SP-D exhibits a cruciform structure consisting of four trimeric subunits. This structural difference causes distinct biological functions. Although L-ficolin was reported to bind to some microorganisms and act as an opsonin, direct inhibition of growth has not been reported. This novel effect of L-ficolin may play an important role in innate immunity in the pulmonary environment, especially via synergistic effects with SP-A.

There are some limitations to this study. First, the ages and sexes of the MAC group did not match those in the control group. Because the control subjects in this study were collected from health check individuals in a hospital, we could not match the status. Since previous reports indicated that the serum concentration of L-ficolin was not affected by age or sex, this mismatch is unlikely to have affected the study’s results [31–33]. We confirmed other 12 samples of age (70.4 ± 5.1 y.o.) and sex (F:M = 10:2) matched samples (data not shown). Mean serum L-ficolin levels were 3.60 ± 0.58 μg/ml, which was no significant difference with this study’s 30 control subjects. L-ficolin levels were not affected by age and sex. Second, the measurement of L-ficolin was taken at a single point in MAC patients; 45.9% of the MAC patients were under treatment or post-treatment. It was not determined whether treatment could change the serum L-ficolin levels. Third, we could not determine whether reduced serum L-ficolin resulted in MAC infection or whether MAC-infected patients lost serum L-ficolin. Several reports indicated that FCN2 gene polymorphisms result in insufficient serum L-ficolin [34, 35]. Single-nucleotide polymorphisms in the promoter region and in exon-8 have significant effects on measured serum levels. Considering this, insufficient L-ficolin could be a risk factor for pulmonary MAC disease.

## Conclusions

In conclusion, insufficient serum L-ficolin is associated with disease presence and extent of pulmonary MAC disease. Serum levels of L-ficolin are a possible biomarker of pulmonary MAC disease.

## Supplementary information

**Additional file 1:Table S1A.** is the summary of HRCT scoring system of the whole lung. To evaluate the disease progression of pulmonary MAC, we scored the HRCT findings. Each score was from 0 to 3 and the score of seven categories were totaled. The average HRCT score of each category was shown in table S1B. The average of total was 6.25 ± 3.30 indicating the patients’ severity were mild to moderate on the HRCT criteria.

## Data Availability

Please contact the corresponding author for data requests.

## Abbreviations

BMI: Body mass index
FC type: Fibrocavitary type
HRCT: High-resolution computed tomography
HU: Hounsfield units
LAM: Lipoarabinomannan
MAC: *Mycobacterium avium* complex
MBL: Mannose-binding lectin;
NB type: Nodular bronchiectatic type
NTM: Nontuberculous mycobacteria
ROC curve: Receiver operating characteristic curve
SP-A and D: Surfactant proteins A and D

## References

[1] Namkoong H, Kurashima A, Morimoto K, Hoshino Y, Hasegawa N, Ato M (2016). Epidemiology of pulmonary nontuberculous mycobacterial disease, Japan. Emerg Infect Dis.

[2] Diel R, Lipman M, Hoefsloot W (2018). High mortality in patients with *Mycobacterium avium* complex lung disease: a systematic review. BMC Infect Dis.

[3] Kartalija M, Ovrutsky AR, Bryan CL, Pott GB, Fantuzzi G, Thomas J (2013). Patients with nontuberculous mycobacterial lung disease exhibit unique body and immune phenotypes. Am J Respir Crit Care Med.

[4] Chan ED, Iseman MD (2010). Slender, older women appear to be more susceptible to nontuberculous mycobacterial lung disease. Gend Med.

[5] Ordway D, Henao-Tamayo M, Smith E, Shanley C, Harton M, Troudt J (2008). Animal model of *Mycobacterium abscessus* lung infection. J Leukoc Biol.

[6] Nishimura T, Fujita-Suzuki Y, Mori M, Carpenter SM, Fujiwara H, Uwamino Y (2016). Middle-aged to elderly women have a higher asymptomatic infection rate with *Mycobacterium avium* complex, regardless of body habitus. Respirology.

[7] Jeon K, Kim S-Y, Jeong B-H, Chang B, Shin SJ, Koh W-J (2013). Severe vitamin D deficiency is associated with non-tuberculous mycobacterial lung disease: a case–control study. Respirology.

[8] Liu Y, Endo Y, Iwaki D, Nakata M, Matsushita M, Wada I, Inoue K (2005). Human M-ficolin is a secretory protein that activates the lectin complement pathway. J Immunol.

[9] Le Y, Lee SH, Kon OL, Lu J (1998). Human L-ficolin: plasma levels, sugar specificity, and assignment of its lectin activity to the fibrinogen-like (FBG) domain. FEBS Lett.

[10] Sugimoto R, Yae Y, Akaiwa M, Kitajima S, Shibata Y, Sato H (1998). Cloning and characterization of the Hakata antigen, a member of the ficolin/opsonin p35 lectin family. J Biol Chem.

[11] Kwon S, Kim MS, Kim D, Lee KW, Choi SY, Park J (2007). Identification of a functionally relevant signal peptide of mouse ficolin A. J Biochem Mol Biol.

[12] Endo Y, Liu Y, Kanno K, Takahashi M, Matsushita M, Fujita T (2004). Identification of the mouse H-ficolin gene as a pseudogene and orthology between mouse ficolins a/B and human L/M-ficolins. Genomics.

[13] Nahid AM, Sugii S (2006). Binding of porcine ficolin-alpha to lipopolysaccharides from gram-negative bacteria and lipoteichoic acids from gram-positive bacteria. Dev Comp Immunol.

[14] Garred P, Genster N, Pilely K, Bayarri-Olmos R, Rosbjerg A, Ma YJ (2016). A journey through the lectin pathway of complement-MBL and beyond. Immunol Rev.

[15] Aoyagi Y, Adderson EE, Min JG, Matsushita M, Fujita T, Takahashi S (2005). Role of L-ficolin/mannose-binding lectin-associated serine protease complexes in the opsonophagocytosis of type III group B streptococci. J Immunol.

[16] Krarup A, Mitchell DA, Sim RB (2008). Recognition of acetylated oligosaccharides by human L-ficolin. Immunol Lett.

[17] Krarup A, Sørensen UB, Matsushita M, Jensenius JC, Thiel S (2005). Effect of capsulation of opportunistic pathogenic bacteria on binding of the pattern recognition molecules mannan-binding lectin, L-ficolin, and H-ficolin. Infect Immun.

[18] Runza VL, Schwaeble W, Männel DN (2008). Ficolins: novel pattern recognition molecules of the innate immune response. Immunobiology.

[19] Endo Y, Takahashi M, Iwaki D, Ishida Y, Nakazawa N, Kodama T (2013). Mice deficient in ficolin, a lectin complement pathway recognition molecule, are susceptible to Streptococcus pneumoniae infection. J Immunol.

[20] Ariki S, Kojima T, Gasa S, Saito A, Nishitani C, Takahashi M, Shimizu T (2011). Pulmonary collectins play distinct roles in host defense against *Mycobacterium avium*. J Immunol.

[21] Kudo K, Sano H, Takahashi H, Kuronuma K, Yokota S, Fujii N (2004). Pulmonary collectins enhance phagocytosis of *Mycobacterium avium* through increased activity of mannose receptor. J Immunol.

[22] Griffith DE, Aksamit T, Brown-Elliott BA, Catanzaro A, Daley C, Gordin F (2007). An official ATS/IDSA statement: diagnosis, treatment, and prevention of nontuberculous mycobacterial diseases. Am J Respir Crit Care Med.

[23] Fowler SJ, French J, Screaton NJ, Foweraker J, Condliffe A, Haworth CS (2006). Nontuberculous mycobacteria in bronchiectasis: prevalence and patient characteristics. Eur Respir J.

[24] Iwamoto Tomotada, Nakajima Chie, Nishiuchi Yukiko, Kato Tomoko, Yoshida Shiomi, Nakanishi Noriko, Tamaru Aki, Tamura Yutaka, Suzuki Yasuhiko, Nasu Masao (2012). Genetic diversity of Mycobacterium avium subsp. hominissuis strains isolated from humans, pigs, and human living environment. Infection, Genetics and Evolution.

[25] Gochi M, Takayanagi N, Kanauchi T, Ishiguro T, Yanagisawa T, Sugita Y (2015). Retrospective study of the predictors of mortality and radiographic deterioration in 782 patients with nodular/bronchiectatic *Mycobacterium avium* complex lung disease. BMJ Open.

[26] Kitada S, Kobayashi K, Ichiyama S, Takakura S, Sakatani M, Suzuki K (2008). Serodiagnosis of *Mycobacterium avium*-complex pulmonary disease using an enzyme immunoassay kit. Am J Respir Crit Care Med.

[27] Luo F, Sun X, Wang Y, Wang Q, Wu Y, Pan Q (2013). Ficolin-2 defends against virulent *Mycobacteria tuberculosis* infection *in vivo*, and its insufficiency is associated with infection in humans. PLoS One.

[28] Chalmers JD, Fleming GB, Rutherford J, Matsushita M, Kilpatrick DC, Hill AT (2014). Serum ficolin-2 in hospitalized patients with community-acquired pneumonia. Inflammation.

[29] Kilpatrick DC, Chalmers JD, MacDonald SL, Murray M, Mohammed A, Hart SP (2009). Stable bronchiectasis is associated with low serum L-ficolin concentrations. Clin Respir J.

[30] Metzger ML, Michelfelder I, Goldacker S, Melkaoui K, Litzman J, Guzman D (2015). Low ficolin-2 levels in common variable immunodeficiency patients with bronchiectasis. Clin Exp Immunol.

[31] Sallenbach S, Thiel S, Aebi C, Otth M, Bigler S, Jensenius JC (2011). Serum concentrations of lectin-pathway components, children and in healthy neonates adults: mannan-binding lectin (MBL), M-, L-, and H-ficolin, and MBL-associated serine protease-2 (MASP-2). Pediatr Allergy Immunol.

[32] Gaya da Costa M, Poppelaars F, van Kooten C, Mollnes TE, Tedesco F, Würzner R (2018). Age and sex-associated changes of complement activity and complement levels in a healthy Caucasian population. Front Immunol.

[33] Troldborg A, Hansen A, Hansen SWK, Jensenius JC, Stengaard-Pedersen K, Thiel S (2016). Lectin complement pathway proteins in healthy individuals. Clin Exp Immunol.

[34] Hummelshoj T, Munthe-Fog L, Madsen HO, Fujita T, Matsushita M, Garred P (2005). Polymorphisms in the FCN2 gene determine serum variation and function of Ficolin-2. Hum Mol Genet.

[35] Cedzynski M, Nuytinck L, Atkinson APM, St Swierzko A, Zeman K, Szemraj J (2007). Extremes of L-ficolin concentration in children with recurrent infections are associated with single nucleotide polymorphisms in the FCN2 gene. Clin Exp Immunol.

