# Supplementary material for: Insufficient serum L-ficolin is associated with disease presence and extent of pulmonary Mycobacterium avium complex disease
Source: Respir Res. 2019 Oct 21;20:224. doi: 10.1186/s12931-019-1185-9 (PMC6805425; doi:10.1186/s12931-019-1185-9)
Supplement: Supplementary file 1 — Additional file 1:Table S1A. is the summary of HRCT scoring system of the whole lung. To evaluate the disease progression of pulmonary MAC, we scored the HRCT findings. Each score was from 0 to 3 and the score of seven categories were totaled. The average HRCT score of each category was shown in table S1B. The average of total was 6.25 ± 3.30 indicating the patients’ severity were mild to moderate on the HRCT criteria. [file 12931_2019_1185_MOESM1_ESM.pptx]

## Slide 1
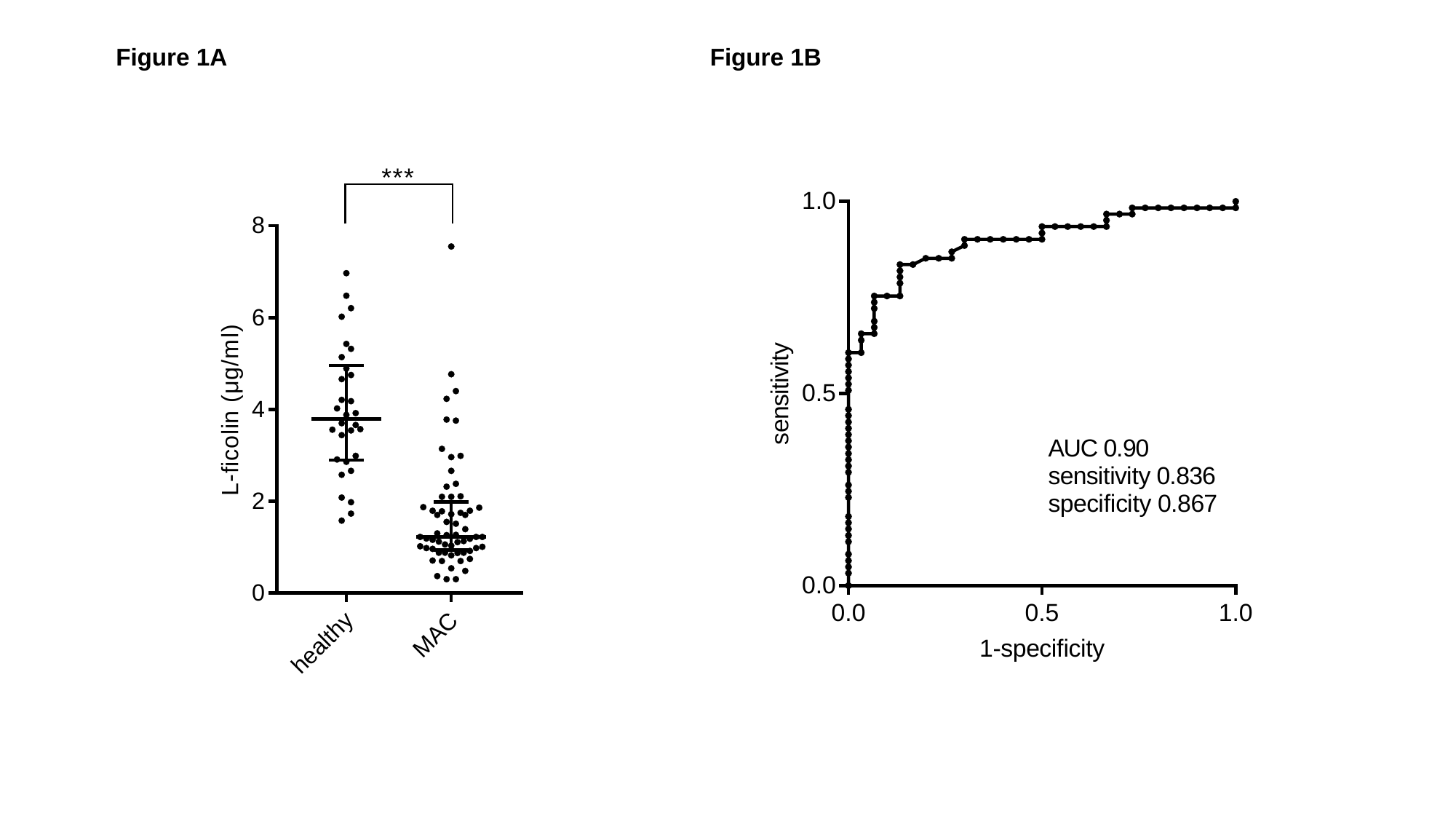

Figure 1A
Figure 1B

## Slide 2
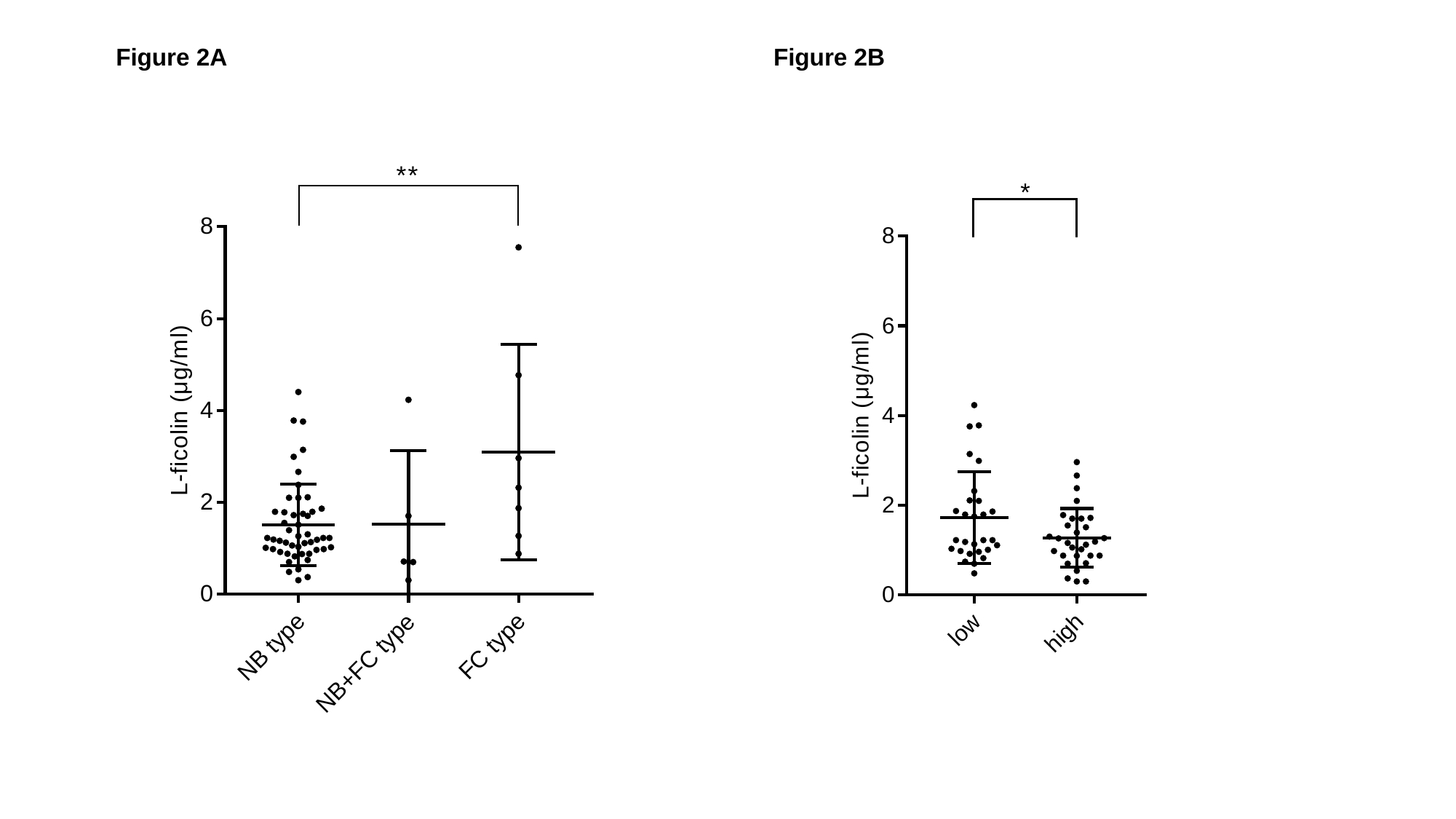

Figure 2A
Figure 2B

## Slide 3
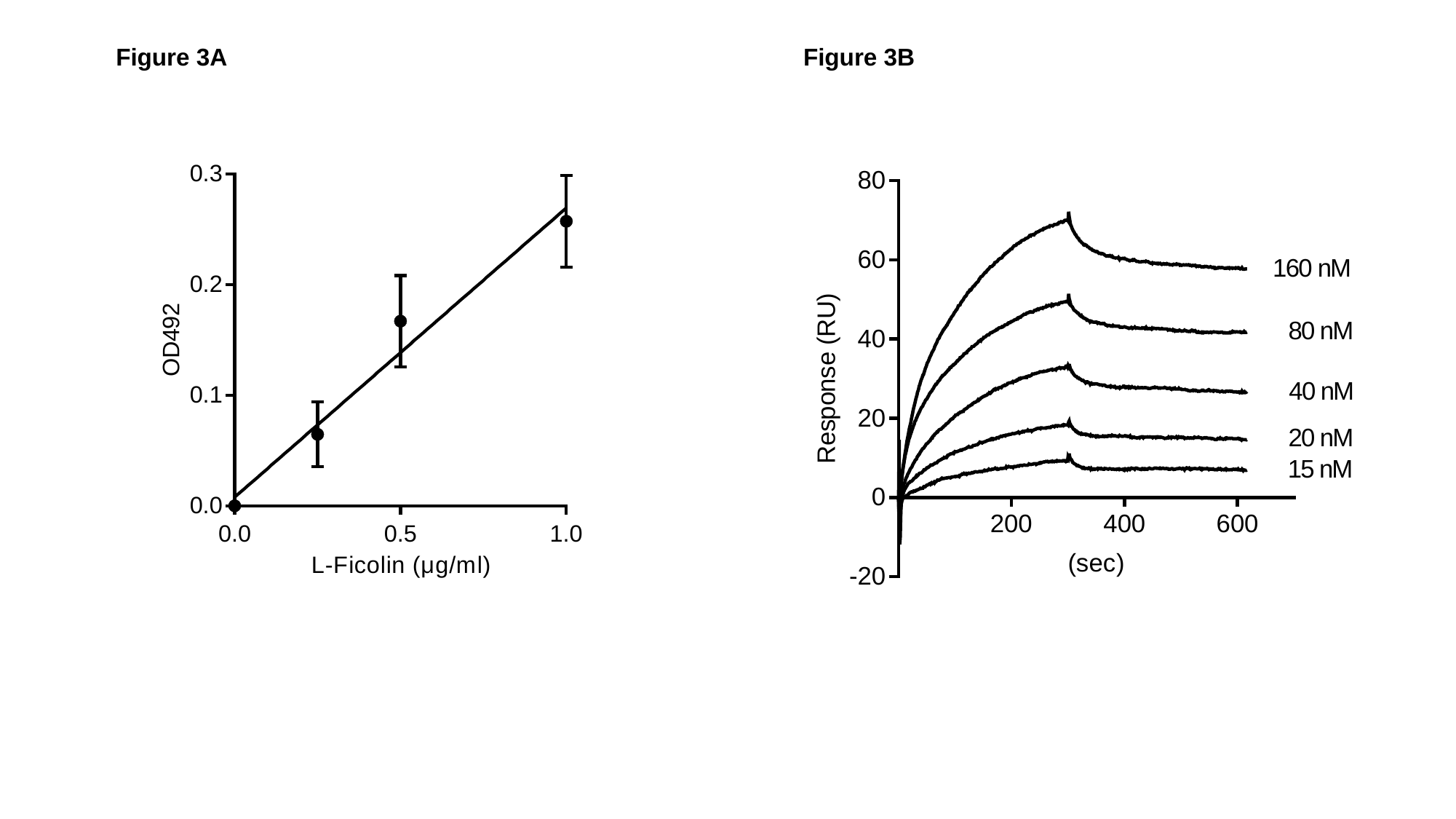

Figure 3A
Figure 3B

## Slide 4
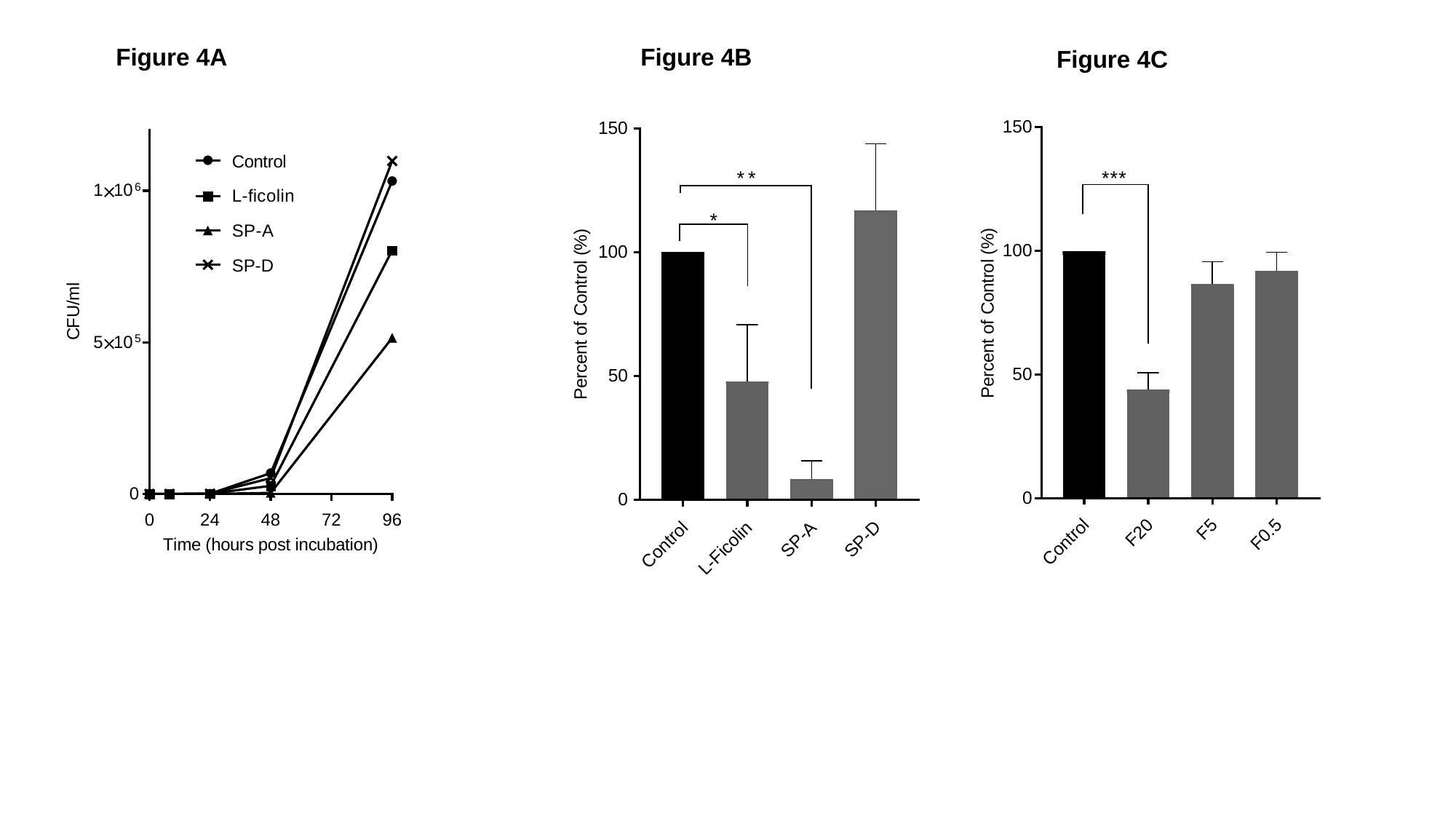

Figure 4A
Figure 4B
Figure 4C
